# Deglaciation explains bat extinction in the Caribbean

**DOI:** 10.1002/ece3.399

**Published:** 2012-11-06

**Authors:** Liliana M Dávalos, Amy L Russell

**Affiliations:** 1 Department of Ecology and Evolution and Consortium for Interdisciplinary Environmental Research, State University of New York at Stony BrookStony Brook, New York, 11794; 2 Department of Biology, Grand Valley State UniversityAllendale, Michigan, 49401

**Keywords:** Climate change, deglaciation, glaciation, sea-level rise, species–area relationship

## Abstract

Ecological factors such as changing climate on land and interspecific competition have been debated as possible causes of postglacial Caribbean extinction. These hypotheses, however, have not been tested against a null model of climate-driven postglacial area loss. Here, we use a new Quaternary mammal database and deep-sea bathymetry to estimate species–area relationships (SARs) at present and during the Last Glacial Maximum (LGM) for bats of the Caribbean, and to model species loss as a function of area loss from rising sea level. Island area was a significant predictor of species richness in the Bahamas, Greater Antilles, and Lesser Antilles at all time periods, except for the Lesser Antilles during the LGM. Parameters of LGM and current SARs were similar in the Bahamas and Greater Antilles, but not the Lesser Antilles, which had fewer estimated species during the LGM than expected given their size. Estimated postglacial species losses in the Bahamas and Greater Antilles were largely explained by inferred area loss from rising sea level in the Holocene. However, there were more species in the Bahamas at present, and fewer species in the smaller Greater Antilles, than expected given island size and the end-Pleistocene/early Holocene SARs. Poor fossil sampling and ecological factors may explain these departures from the null. Our analyses illustrate the importance of changes in area in explaining patterns of species richness through time and emphasize the role of the SAR as a null hypothesis in explorations of the impact of novel ecological interactions on extinction.

## Introduction

The terrestrial mammal fauna of the West Indies once comprised sloths, primates, rodents, insectivores, and bats (Morgan and Woods 1986; Dávalos 2004). During the late Pleistocene and early Holocene waves of extinction nearly obliterated this biota, but the majority of the bats survived (Dávalos and Turvey 2012). Bats were not traditionally hunted for food in the Caribbean, and many species have proven resilient in the face of introduced predators (although see Tejedor et al. 2005). Although habitat changes (Pregill and Olson 1981) and competition (Koopman and Williams 1951; Williams 1952) have been proposed to explain extirpations of Caribbean bats since the Last Glacial Maximum (LGM), sea-level rise caused by nonanthropogenic climate change may be a more important driver of extinction in this fauna (Morgan 2001; Dávalos and Turvey 2012).

The most drastic climatic change since the late Pleistocene was the shift from the conditions of the LGM – from ∼22,000 to ∼19,000 years before present (yBP; Yokoyama et al. 2000) – to the interglacial climate prevalent since the mid-Holocene. In the terrestrial ecosystems of the West Indies, deglaciation replaced xerophytic environments with mesic habitats (Higuera-Gundy et al. 1998; White et al. 1998; Pajón et al. 2001; McFarlane et al. 2002). One key consequence of climate change was sea-level rise. From 15,000 to 7000 yBP, sea level rose from 100 to 10 m below current level in three bursts marking the collapse of ice sheets, the reorganization of ocean–atmosphere circulation, and the release of glacial meltwater (Blanchon and Shaw 1995). This period corresponds to the inferred last occurrences of many bats, as well as birds and lizards, on many islands (Pregill and Olson 1981; Morgan and Woods 1986; Morgan 1989, 1994, 2001; McFarlane et al. 2002). There are no direct fossil dates for extinct bat populations, and the 22,000- to 7000-yBP interval corresponding to dramatic rises in sea level overlaps with all indirect radiometric dates for extinct bat populations (Suárez and Díaz-Franco 2003; Jiménez Vázquez et al. 2005). Here, the considerable island area loss caused by deglaciation during the end-Pleistocene/early Holocene serves as an abiotic null hypothesis to explain extinction patterns in the absence of more recent ecological changes, including anthropogenic species introductions, habitat, and climate change.

**Figure 1:**
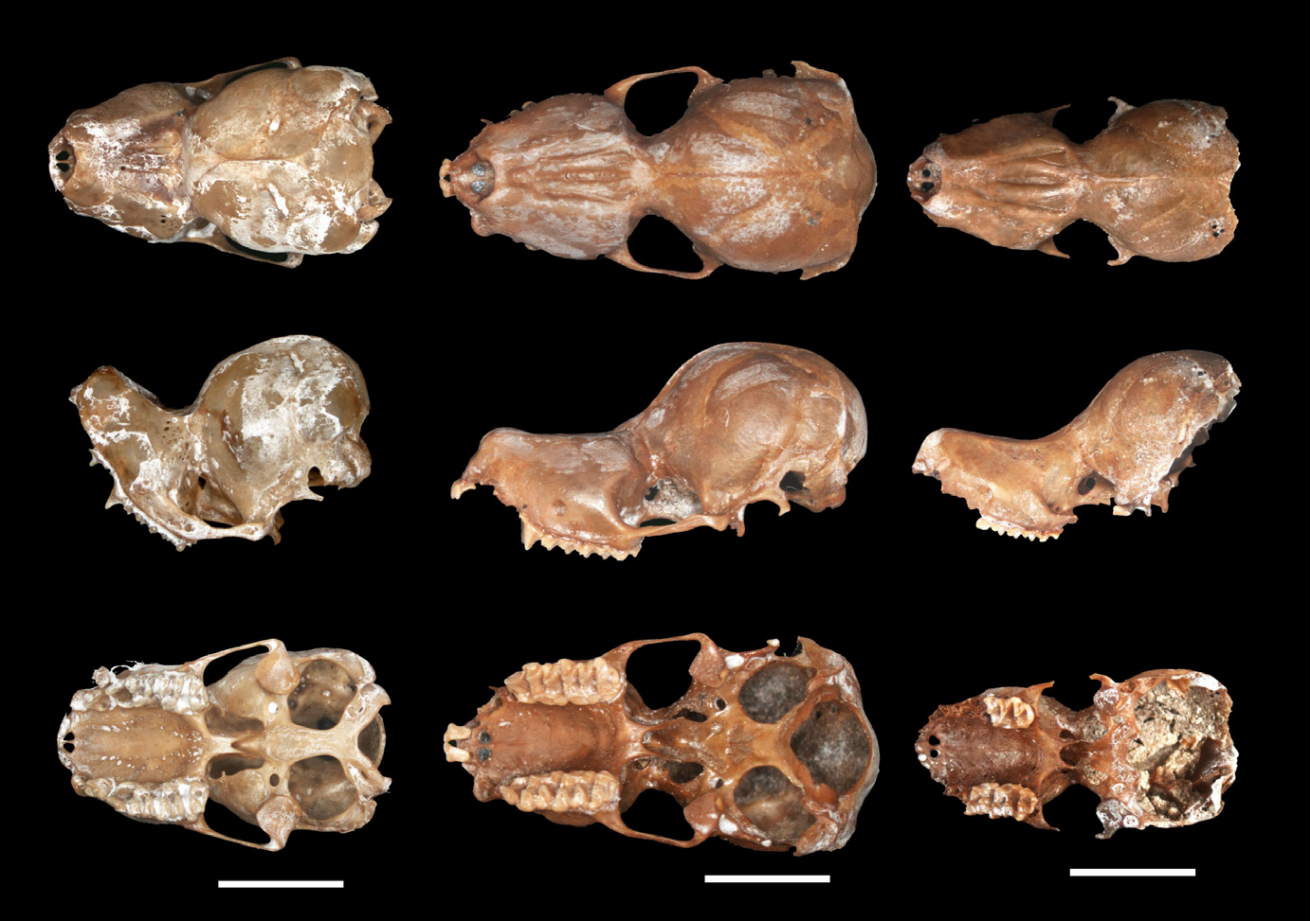
Representative subfossils (Chiroptera: Mormoopidae) from a cave deposit in the Dominican Republic. From left: *Mormoops blainvillei*, *Pteronotus parnellii*, and *P. quadridens*. White bar indicates 1 cm. Quaternary fossils and subfossils on many islands of the West Indies enable estimates of species richness at the Last Glacial Maximum, before sea-level rise drastically reduced the area of most islands.

We combine analyses of bathymetry and estimates of bat species richness across three Caribbean archipelagos to estimate land area and species richness at the LGM (before the end-Pleistocene/early Holocene area loss) and quantify the impact of area declines on bat species richness. The bat biota of the Caribbean is uniquely suited to evaluate the species–area relationship (SAR) across time: the land area experienced significant changes since the LGM, and numerous bat fossils in cave sediments enable reasonable estimates of species richness at the end of the Pleistocene (Fig. 1). In addition, the Caribbean has experienced the highest level of recent species loss of any mammal fauna in the world (MacPhee and Flemming 1999; Morgan 2001; MacPhee 2009; Turvey 2009), so we expect these data will retain considerable power to examine the effects of recent extinction.

## Material and Methods

At the LGM, sea levels were 120–135 m below current level (Hearty 1998; Clark et al. 2003). To estimate the area of the islands at the LGM, we decreased sea level by 125 m on the global 1-km grid topography and bathymetry of Becker and Sandwell (2008) in Lambert cylindrical equal-area projection. We investigated the sensitivity of the LGM area estimate for the Bahamas to coral accretion by estimating the effect of a linear growth rate of 1 cm/year over the last 20,000 yBP (Johnson and Pérez 2006). The resulting linear change (200 m) was subtracted from the radius of individual Bahamian banks, and the corresponding areas were recalculated. Current areas were calculated based on current sea level, or compiled from the United Nations Environment Program Earthwatch Database (http://islands.unep.ch/Tiarea.htm).

To obtain species richness, we used the extant and extinct mammalian distribution database for the islands of the Caribbean (Willig et al. 2010; Dávalos and Turvey 2012). Species richness at the LGM was calculated as the sum of current and extinct species richness. Stratigraphic and indirect radiometric analyses of fossil faunas including bats have found Late Wisconsinan or Early Holocene dates for the remains (Koopman and Williams 1951; Morgan 2001; McFarlane et al. 2002; Suárez and Díaz-Franco 2003; Mancina and Garcia-Rivera 2005; Steadman et al. 2007), indicating most fossil populations would have been extant at the LGM. The ∼7000 yBP date for a Cuban fauna of Jiménez Vázquez et al. (2005) coincides with the date at which sea level reached ∼10 m below present levels (Blanchon and Shaw 1995). Stratigraphic and radiometric analyses support end-Pleistocene/early Holocene dates for included fossil species, and modern faunal surveys strongly support our designation of species as extinct. The only species in the current fauna thought to have immigrated so recently that it may not have been part of the end-Pleistocene/early Holocene fauna is *Artibeus jamaicensis* (Koopman and Williams 1951; Williams 1952; Morgan 1994), so we estimated SARs with and without this species to assess its effect on results.

Based on biogeographic and geological similarities, we subdivided analyses into three archipelagos: the Bahamas, the Greater Antilles, and the Lesser Antilles (Willig et al. 2010). The fauna of Trinidad, Tobago, Margarita, Aruba, Bonaire, and Curaçao were excluded because these islands are characterized by a South American bat biota (Morgan and Woods 1986; Koopman 1989; Morgan 2001) and are likely subject to fundamentally different biogeographic processes.

To estimate the parameters of the SARs, we fitted separate linear models of species as a function of area for the LGM and the present. The slope of the SAR is expected to become steeper with increasing isolation (MacArthur and Wilson 1967); therefore, higher sea levels since the LGM may have shifted the slope of the current curve relative to the past. Comparisons between the predictions based on the SAR at the LGM and current observations would not be valid if that were the case. To test for homogeneity of slopes (*z*), we fitted analysis of covariance (ANCOVA) models of species as a function of area (both log-transformed) with LGM or current islands as the factor. These models also tested the homogeneity of the intercept term of SARs – log(*c*) – through time.

Since:


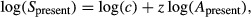


and


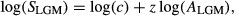


assuming c and z remain constant – tested as above – then:


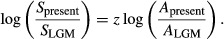


Based on this relationship between changes in richness and area, we modeled log-transformed ratios of present/LGM richness as a function of the ratio of areas without an intercept term.

Finally, we compared the predicted species diversity of each island based on the LGM SAR to the observed current species diversity. If the LGM-based SAR correctly estimated current richness, then islands should fall along a curve of slope = 1 in a plot of predicted versus observed richness. The area below the expected line would indicate underestimated species richness at the LGM and/or more species today than predicted. Conversely, the area above the line would indicate fewer species observed today than expected given the LGM SAR. All analyses were conducted in the R v.1.14.2 statistical environment (R Development Core Team 2010).

## Results

**Figure 2:**
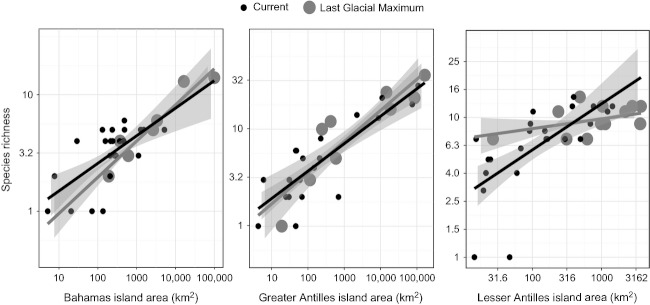
Species–area curves for three Caribbean archipelagos at the Last Glacial Maximum (LGM) and present. Shaded areas indicate the 95% confidence interval around the mean of the curves. LGM species–area relationships (SARs) were highly significant for the Bahamas and the Greater Antilles, but not the Lesser Antilles (Table 1). Current SARs were highly significant for all archipelagos (Table 1). The slopes of the curves fitted for each time period were not statistically different in the Bahamas or Greater Antilles, but were significantly different in the Lesser Antilles (Table 2).

Island area was a significant predictor of species richness for all archipelagos and time periods, excluding the Lesser Antilles at the LGM (Table 1, Fig. 2). Species–area curves for the Bahamas and the Greater Antilles had similar slopes for the LGM and present (Table 2). In contrast, the species–area curves fitted for the two time periods for the Lesser Antilles had significantly different slopes, with LGM area explaining a very small portion of the variation in richness at the LGM compared with the present relationship (Tables 1 and 2). We excluded this archipelago from estimates of species loss as a function of area loss, and from comparisons of LGM SARs to present richness because of the heterogeneity of slopes of LGM and current SARs (Table 2).

**Table 1:** Slopes and significance of species–area relationships for Caribbean archipelagos

Archipelago	Period	Slope ± standard error	*R*^2^	*P*-value
Bahamas	Last Glacial Maximum (LGM)	0.33 ± 0.04	0.88	0.0003
	Present	0.24 ± 0.06	0.40	0.0007
	Present/LGM	0.27 ± 0.02	0.83	0.0000
Greater Antilles	LGM	0.32 ± 0.06	0.77	0.0012
	Present	0.28 ± 0.04	0.69	0.0000
	Present/LGM	0.28 ± 0.04	0.85	0.0000
Lesser Antilles	LGM	0.08 ± 0.04	0.15	0.1076
	Present	0.33 ± 0.07	0.44	0.0003

**Table 2:** Analyses of covariance (ANCOVA) testing for the homogeneity of intercepts and slopes of species–area relationships at present and Last Glacial Maximum

Archipelago	Time period as factor	*P*-value	Interaction log area and time period	*P*-value
Bahamas	0.267 ± 0.300	0.381	−0.074 ± 0.094	0.441
Greater Antilles	0.093 ± 0.243	0.705	−0.038 ± 0.074	0.611
Lesser Antilles	−0.672 ± 0.308	0.037	0.260 ± 0.112	0.027

**Figure 3:**
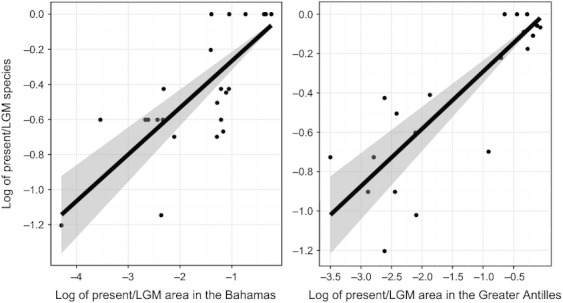
Curves for change in species richness from the Last Glacial Maximum (LGM) to the present as a function of change in area in two Caribbean archipelagos. Shaded areas indicate the 95% confidence interval around the mean of the curves. All relationships were highly significant (Table 1).

**Figure 4:**
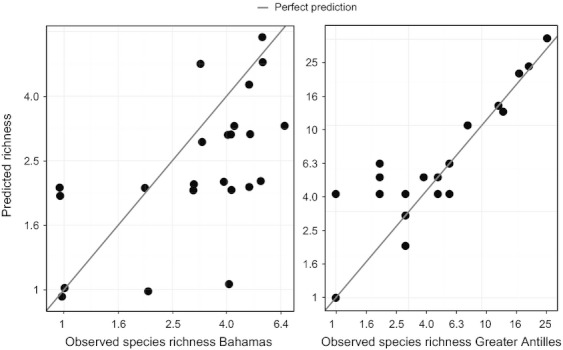
Predicted versus observed species richness in the Bahamas and Greater Antilles. The curve of slope = 1 indicates where the Last Glacial Maximum (LGM) species–area relationships (SAR) perfectly predicts current species richness. The LGM SAR underestimates current species richness in the area below the curve and overestimates current richness in the area above the curve.

Island size change since the LGM explained most, but not all, of the decline in species richness on the Bahamas and Greater Antilles (Table 2, Fig. 3). To examine the relationship between LGM and current SARs, we used LGM SARs to predict current species richness from current island area (Fig. 4). If SARs have not changed since the Pleistocene, then LGM SARs should predict observed species richness, and a plot of observed and predicted species richness should show islands roughly falling along an expected line of slope = 1. In the majority of islands in the Bahamas, the LGM SAR predicted fewer species at present than have been observed. The opposite was true for the Greater Antilles, where most of the significant deviations from the expected relationship involved smaller islands with lower-than-expected current species richness.

Species richness on all archipelagos may have changed because of colonization, and island area in the Bahamas may have increased from coral accretion. Widespread species shared with the continent and lacking fossil records are the most likely recent colonizers. Only *Artibeus jamaicensis* meets these criteria: it may be a recent colonizer in the Bahamas. This species was inferred to be present in every island bank of the Greater and Lesser Antilles, so its exclusion cannot change the slope of those SARs. We conducted analyses accounting for coral accretion and excluding *Artibeus jamaicensis* from the Bahamas (Supporting information). The area difference when accounting for coral deposition in Bahamian banks since the LGM ranged from 0.2% to 5.1% of the estimated LGM area, with a median of 1.3%, and a mean of 2.0%. Over the timespan considered here, colonization by new species has had minimal effect on species richness. Therefore, analyses presented in the main text ignored coral accretion and included *A. jamaicensis* in the LGM Bahamian fauna.

## Discussion

We find that island size change is the greatest single predictor of species loss in the Bahamas and Greater Antilles. Although this abiotic change in island area explains most of the observed species loss, there are more species in the Bahamas, and fewer in the smaller Greater Antilles, than expected given current island sizes and predictions from LGM SARs. In the Lesser Antilles, however, there are fewer species known from the LGM than were expected given their size.

### Species–area relationships in the Lesser Antilles

Island area was not a significant predictor of species richness at the LGM in the Lesser Antilles (Table 1). This result could arise by overestimating the LGM richness of smaller islands that were only recently colonized, or underestimating the richness of larger islands whose fossil records may be incomplete, or both. If the high richness of the smallest island bank (Saba) drove this result, then removing this point would result in a steeper, significant relationship, but it does not (recalculated slope 0.04 ± 0.06, linear model *P*-value = 0.5210). Several island banks larger than 1500 km^2^ share similar richness estimates of ∼10 despite differences of hundreds of km^2^ in area at the LGM. The expected species richness for these island banks is at least 16 species based on the current curve (Fig. 2). Despite their large size at the LGM, the estimated species richness of these banks is small, and it is likely underestimated because of the scant fossil record of this archipelago. Few fossil sites in the Lesser Antilles have been excavated, and only on Anguilla, and Antigua and Barbuda (these last two islands are part of the same bank; Morgan 2001). The small number of documented fossil species explains the independence of richness from area in LGM estimates for this archipelago. Our results suggest that more fossil species remain to be discovered from the late Pleistocene/early Holocene of the Lesser Antilles.

### Area loss explains most of the change in richness in the Bahamas and Greater Antilles

Five hypotheses other than overhunting and predators introduced by humans have been proposed to explain Caribbean mammal extinction events since the LGM: (1) postglacial sea-level rise reducing island area (Morgan 2001; Dávalos and Turvey 2012); (2) postglacial sea-level rise flooding caves (Morgan 2001); (3) postglacial climate change replacing xerophytic environments with mesic habitats (Pregill and Olson 1981); (4) competition from new colonizers leading to faunal replacement (Koopman and Williams 1951; Williams 1952), and (5) habitat conversion for human agriculture over the last few thousand years (Gannon et al. 2005). Our estimates of the impact of sea-level change on this biota support the first hypothesis: area loss from postglacial sea-level rise was a major predictor of species loss (Table 1). These results held, even after accounting for sources of error such as coral accretion and the possible recent arrival of *Artibeus jamaicensis* onto the islands (Tables S1 and S2). This model of extinction caused by area loss associated with postglacial sea-level rise has been supported for other Caribbean mammals, such as the giant hutia *Amblyrhiza* in the Sangamonian (McFarlane et al. 1998). We propose extinction caused by area loss as the null hypothesis in investigating insular postglacial extinctions.

In most islands of the Bahamas, LGM SARs predict fewer species at present than are observed. These results could arise through underestimation of species richness at the LGM and suggest that our understanding of the fossil bat biota is incomplete for these banks. A similar analysis of the Greater Antilles showed that SARs for the most species-rich islands in this archipelago are largely unchanged from the LGM (Fig. 4). In smaller islands of the Greater Antilles, however, LGM SARs predict greater species richness than observed. This pattern may be caused by underestimation of current species richness on smaller banks, or because of drivers of richness beyond island area. If current richness at smaller banks were underestimated, then SARs would show a break between smaller and larger areas, with higher slopes at the lower end of the relationship. To evaluate this prediction, we fitted segmented regression models with a single breakpoint for each archipelago (Muggeo 2008), but found no significant breakpoints in the Greater Antillean SAR (*P*-value = 0.189).

Because underestimation on smaller Greater Antillean banks did not explain the lower-than-expected species richness at present, we suggest that alternative ecological explanations such as the collapse of specific habitats (caves), competition, or habitat loss need to be explored.

By accounting for the major effect of area loss on species declines across most of the Caribbean, and highlighting departures from SAR arising from a poor understanding of the fossil bat fauna in the Lesser Antilles and Bahamas, our analyses illuminate the potential scope of ecological constraints, species interactions, and anthropogenic change on the regional Caribbean fauna.

## Supplementary material

**Figure S1.** Species-area curves and observed versus predicted richness for the Bahamas at the LGM and present after excluding *Artibeus jamaicensis* and accounting for coral accretion since the LGM. Shaded areas indicate the 95% confidence interval around the mean of the curves. Left: SARs fitted to observed current and estimated LGM values. Right: predicted versus observed species richness. The curve of slope = 1 indicates where the LGM SAR perfectly predicts current species richness. The LGM SAR underestimates current species richness in the area below the curve, and overestimates current richness in the area above the curve.

**Table S1.** Caribbean bat species inventory by island and archipelago.

**Table S2.** Slopes and significance of SARs for the Bahamas after excluding *Artibeus jamaicensis* and accounting for coral accretion since the LGM.

**Table S3.** Analyses of covariance (ANCOVA) testing for the homogeneity of intercepts and slopes of SARs at LGM and present for the Bahamas after excluding *Artibeus jamaicensis* and accounting for coral accretion since the LGM.
