# Supplementary figures and images for: Deglaciation explains bat extinction in the Caribbean
Source: Ecol Evol. 2012 Nov 6;2(12):3045–51. doi: 10.1002/ece3.399 (PMC3538999; doi:10.1002/ece3.399)

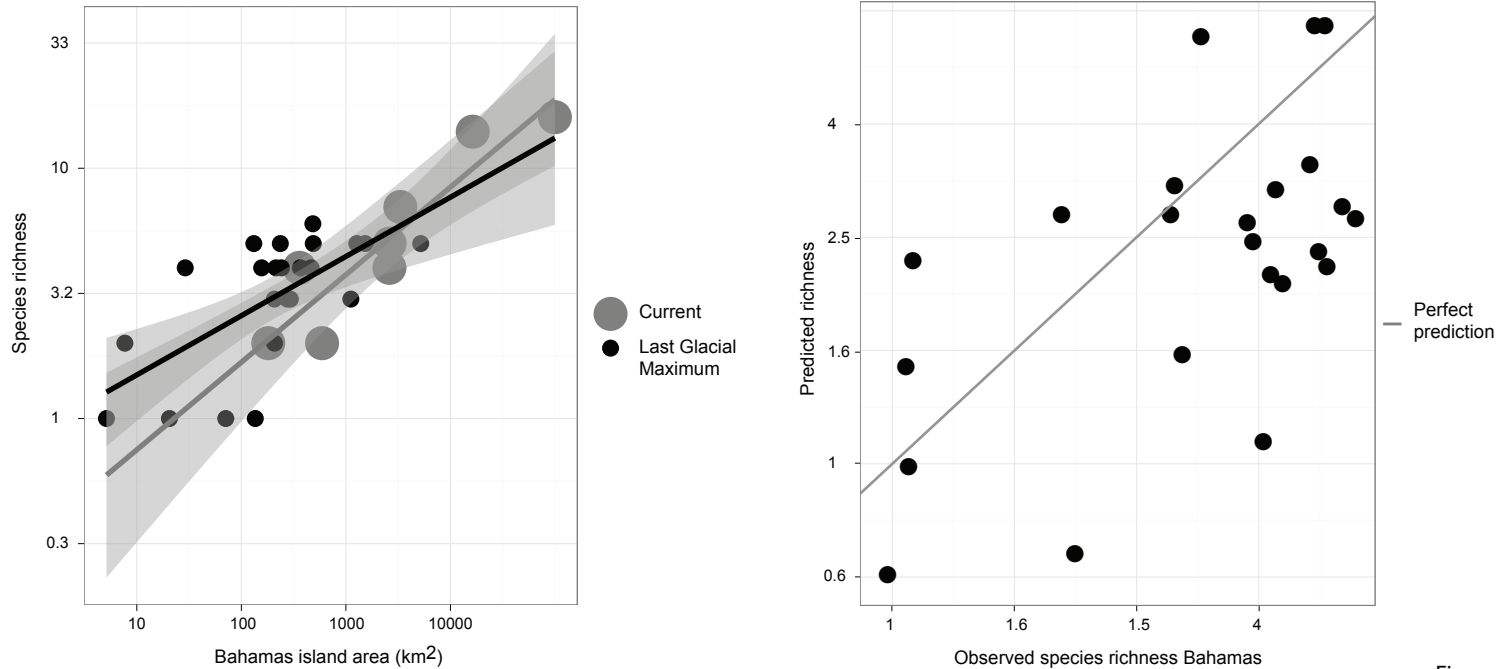

Figure S4

Supplement: Supplementary file 1 [file ece30002-3045-SD1.pdf]

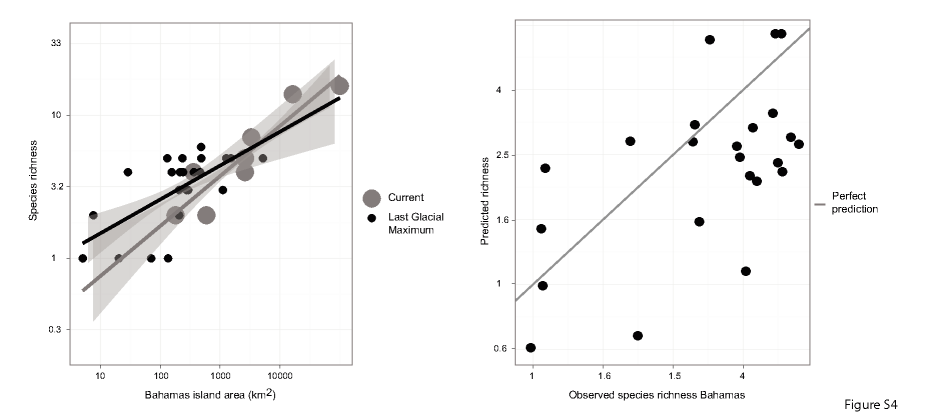

Supplement: Supplementary file 2 [file ece30002-3045-SD2.png]

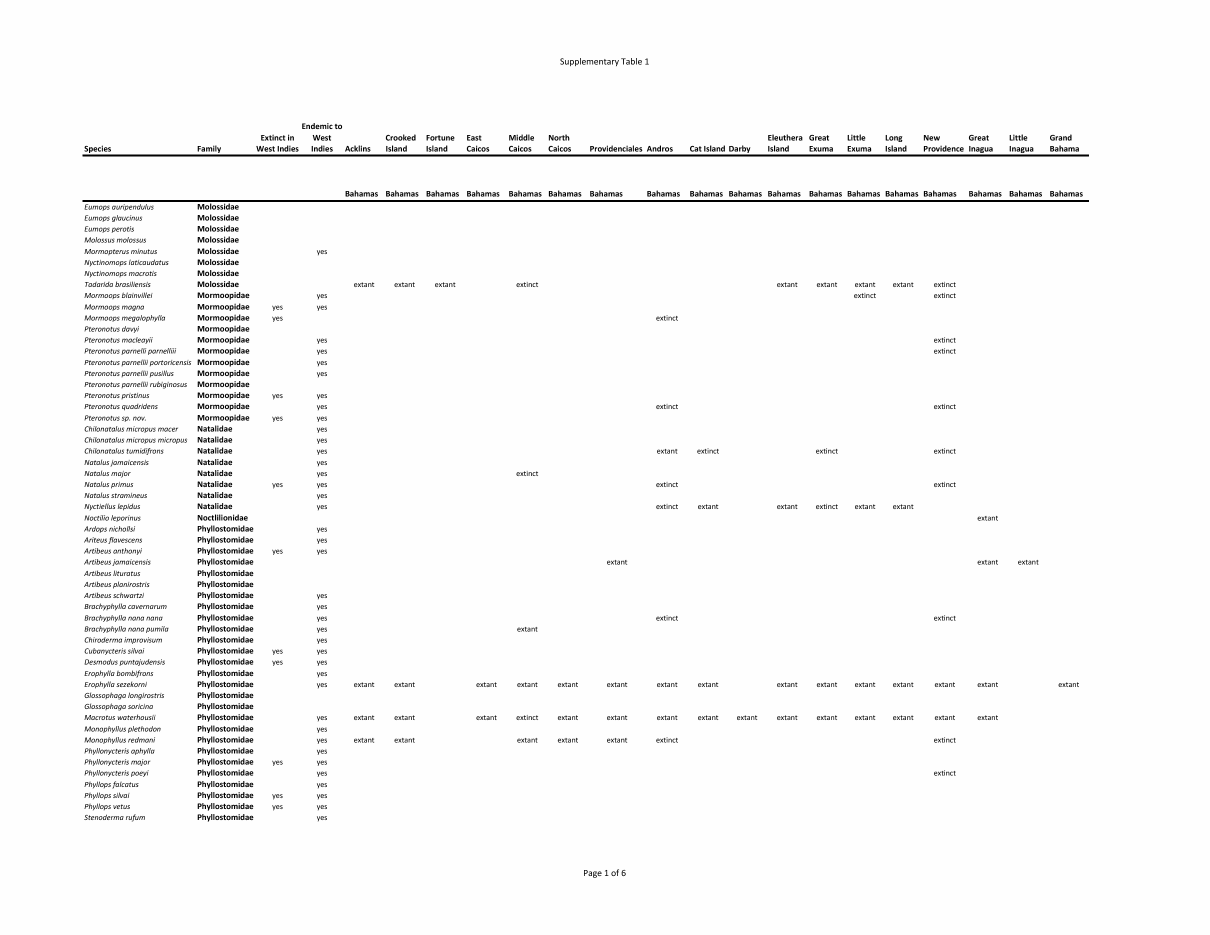

Supplement: Supplementary file 4 [file ece30002-3045-SD4.png]
