# Supplementary material for: Deglaciation explains bat extinction in the Caribbean
Source: Ecol Evol. 2012 Nov 6;2(12):3045–51. doi: 10.1002/ece3.399 (PMC3538999; doi:10.1002/ece3.399)
Supplement: Supplementary file 3 [file ece30002-3045-SD3.pdf]

Species	Family	Extinct in West Indies	Endemic to West Indies	Acklins	Crooked Island	Fortune Island	East Caicos	Middle Caicos	North Caicos	Providenciales	Andros	Cat Island	Darby	Eleuthera Island	Great Exuma	Little Exuma	Long Island	New Providence	Great Inagua	Little Inagua	Grand Bahama
				**Bahamas**	**Bahamas**	**Bahamas**	**Bahamas**	**Bahamas**	**Bahamas**	**Bahamas**	**Bahamas**	**Bahamas**	**Bahamas**	**Bahamas**	**Bahamas**	**Bahamas**	**Bahamas**	**Bahamas**	**Bahamas**	**Bahamas**	**Bahamas**
Eumops auripendulus	**Molossidae**																				
Eumops glaucinus	**Molossidae**																				
Eumops perotis	**Molossidae**																				
Molossus molossus	**Molossidae**																				
Mormopterus minutus	**Molossidae**		yes																		
Nyctinomops laticaudatus	**Molossidae**																				
Nyctinomops macrotis	**Molossidae**																				
Tadarida brasiliensis	**Molossidae**			extant	extant	extant		extinct						extant	extant	extant	extant	extinct			
Mormoops blainvillei	**Mormoopidae**		yes													extinct		extinct			
Mormoops magna	**Mormoopidae**	yes	yes																		
Mormoops megalophylla	**Mormoopidae**	yes									extinct										
Pteronotus davyi	**Mormoopidae**																				
Pteronotus macleayii	**Mormoopidae**		yes															extinct			
Pteronotus parnelli parnellii	**Mormoopidae**		yes															extinct			
Pteronotus parnellii portoricensis	**Mormoopidae**		yes																		
Pteronotus parnellii pusillus	**Mormoopidae**		yes																		
Pteronotus parnellii rubiginosus	**Mormoopidae**																				
Pteronotus pristinus	**Mormoopidae**	yes	yes																		
Pteronotus quadridens	**Mormoopidae**		yes								extinct							extinct			
Pteronotus sp. nov.	**Mormoopidae**	yes	yes																		
Chilonatalus micropus macer	**Natalidae**		yes																		
Chilonatalus micropus micropus	**Natalidae**		yes																		
Chilonatalus tumidifrons	**Natalidae**		yes								extant	extinct			extinct			extinct			
Natalus jamaicensis	**Natalidae**		yes																		
Natalus major	**Natalidae**		yes					extinct													
Natalus primus	**Natalidae**	yes	yes								extinct							extinct			
Natalus stramineus	**Natalidae**		yes																		
Nyctiellus lepidus	**Natalidae**		yes								extinct	extant		extant	extinct	extant	extant				
Noctilio leporinus	**Noctlilionidae**																		extant		
Ardops nichollsi	**Phyllostomidae**		yes																		
Ariteus flavescens	**Phyllostomidae**		yes																		
Artibeus anthonyi	**Phyllostomidae**	yes	yes																		
Artibeus jamaicensis	**Phyllostomidae**									extant									extant	extant	
Artibeus lituratus	**Phyllostomidae**																				
Artibeus planirostris	**Phyllostomidae**																				
Artibeus schwartzi	**Phyllostomidae**		yes																		
Brachyphylla cavernarum	**Phyllostomidae**		yes																		
Brachyphylla nana nana	**Phyllostomidae**		yes								extinct							extinct			
Brachyphylla nana pumila	**Phyllostomidae**		yes					extant													
Chiroderma improvisum	**Phyllostomidae**		yes																		
Cubanycteris silvai	**Phyllostomidae**	yes	yes																		
Desmodus puntajudensis	**Phyllostomidae**	yes	yes																		
Erophylla bombifrons	**Phyllostomidae**		yes																		
Erophylla sezekorni	**Phyllostomidae**		yes	extant	extant		extant	extant	extant	extant	extant	extant		extant	extant	extant	extant	extant	extant		extant
Glossophaga longirostris	**Phyllostomidae**																				
Glossophaga soricina	**Phyllostomidae**																				
Macrotus waterhousii	**Phyllostomidae**		yes	extant	extant		extant	extinct	extant	extant	extant	extant	extant	extant	extant	extant	extant	extant	extant		
Monophyllus plethodon	**Phyllostomidae**		yes																		
Monophyllus redmani	**Phyllostomidae**		yes	extant	extant			extant	extant	extant	extinct							extinct			
Phyllonycteris aphylla	**Phyllostomidae**		yes																		
Phyllonycteris major	**Phyllostomidae**	yes	yes																		
Phyllonycteris poeyi	**Phyllostomidae**		yes															extinct			
Phyllops falcatus	**Phyllostomidae**		yes																		
Phyllops silvai	**Phyllostomidae**	yes	yes																		
Phyllops vetus	**Phyllostomidae**	yes	yes																		
Stenoderma rufum	**Phyllostomidae**		yes																		

Species	Family	Extinct in West Indies	Endemic to West Indies	Acklins	Crooked Island	Fortune Island	East Caicos	Middle Caicos	North Caicos	Providenciales	Andros	Cat Island	Darby	Eleuthera Island	Great Exuma	Little Exuma	Long Island	New Providence	Great Inagua	Little Inagua	Grand Bahama
				**Bahamas**	**Bahamas**	**Bahamas**	**Bahamas**	**Bahamas**	**Bahamas**	**Bahamas**	**Bahamas**	**Bahamas**	**Bahamas**	**Bahamas**	**Bahamas**	**Bahamas**	**Bahamas**	**Bahamas**	**Bahamas**	**Bahamas**	**Bahamas**
Sturnira lilium	**Phyllostomidae**																				
Sturnira thomasi	**Phyllostomidae**		yes																		
Tonatia saurophila	**Phyllostomidae**	yes																			
Antrozous pallidus	**Vespertilionidae**																				
Eptesicus fuscus	**Vespertilionidae**			extant	extant						extant				extant		extant	extant			extant
Eptesicus guadeloupensis	**Vespertilionidae**		yes																		
Lasiurus degelidus	**Vespertilionidae**		yes																		
Lasiurus insularis	**Vespertilionidae**		yes																		
Lasiurus intermedius	**Vespertilionidae**		yes																		
Lasiurus minor	**Vespertilionidae**		yes							extant	extant	extant					extant	extant	extant		extant
Lasiurus pfeifferi	**Vespertilionidae**		yes																		
Myotis cf. M. austroriparius	**Vespertilionida(**	yes	yes																		
Myotis dominicensis	**Vespertilionidae**		yes																		
Myotis martiniquensis	**Vespertilionidae**		yes																		
Nycticeius cubanus	**Vespertilionidae**		yes																		
			**Extant # species**	**5**	**5**	**1**	**2**	**3**	**3**	**5**	**5**	**4**	**1**	**4**	**4**	**4**	**6**	**4**	**5**	**1**	**3**
			**Extinct # species**	**0**	**0**	**0**	**0**	**3**	**0**	**0**	**6**	**1**	**0**	**0**	**2**	**1**	**0**	**10**	**0**	**0**	**0**
			**Total species**	**5**	**5**	**1**	**2**	**6**	**3**	**5**	**11**	**5**	**1**	**4**	**6**	**5**	**6**	**14**	**5**	**1**	**3**

Species	Family	Extinct in West Indies	Endemic to West Indies	Great Abaco	Little Abaco	Mayaguana	East Plana Cay	San Salvador	Cayman Brac	Little Cayman	Cuba	Isle of Pines	Grand Cayman	Hispaniola	Ile de la Gonave	Ile de la Tortue	Jamaica	Mona	Navassa	Anegada	Culebra	Guana	Puerto Rico	St. John
				Bahamas	Bahamas	Bahamas	Bahamas	Bahamas	Greater Antilles	Greater Antilles	Greater Antilles	Greater Antilles	Greater Antilles	Greater Antilles	Greater Antilles	Greater Antilles	Greater Antilles	Greater Antilles	Greater Antilles	Greater Antilles	Greater Antilles	Greater Antilles	Greater Antilles	Greater Antilles
Eumops auripendulus	**Molossidae**																extant							
Eumops glaucinus	**Molossidae**										extant						extant							
Eumops perotis	**Molossidae**										extant													
Molossus molossus	**Molossidae**								extant		extant	extant	extant	extant	extant		extant				extant	extant	extant	extant
Mormopterus minutus	**Molossidae**		yes								extant													
Nyctinomops laticaudatus	**Molossidae**										extant													
Nyctinomops macrotis	**Molossidae**										extant			extant			extant							
Tadarida brasiliensis	**Molossidae**			extant	extant						extant	extant	extant	extant			extant						extant	extant
Mormoops blainvillei	**Mormoopidae**		yes	extinct							extant			extant	extinct		extant	extant					extant	
Mormoops magna	**Mormoopidae**	yes	yes								extinct													
Mormoops megalophylla	**Mormoopidae**	yes		extinct							extinct			extinct			extinct							
Pteronotus davyi	**Mormoopidae**																							
Pteronotus macleayii	**Mormoopidae**		yes								extant	extant					extant							
Pteronotus parnelli parnellii	**Mormoopidae**		yes	extinct							extant	extinct	extinct				extant							
Pteronotus parnellii portoricensis	**Mormoopidae**		yes															extant					extant	
Pteronotus parnellii pusillus	**Mormoopidae**		yes											extant	extinct									
Pteronotus parnellii rubiginosus	**Mormoopidae**																							
Pteronotus pristinus	**Mormoopidae**	yes	yes								extinct													
Pteronotus quadridens	**Mormoopidae**		yes	extinct							extant			extant			extant						extant	
Pteronotus sp. nov.	**Mormoopidae**	yes	yes											extinct										
Chilonatalus micropus macer	**Natalidae**		yes								extant	extant	extinct											
Chilonatalus micropus micropus	**Natalidae**		yes											extant			extant							
Chilonatalus tumidifrons	**Natalidae**		yes	extant				extant																
Natalus jamaicensis	**Natalidae**		yes														extant							
Natalus major	**Natalidae**		yes											extant										
Natalus primus	**Natalidae**	yes	yes	extinct							extant	extinct	extinct											
Natalus stramineus	**Natalidae**		yes																					
Nyctiellus lepidus	**Natalidae**		yes								extant	extant												
Noctilio leporinus	**Noctlilionidae**										extant	extant		extant			extant	extant			extant		extant	extant
Ardops nichollsi	**Phyllostomidae**		yes																					
Ariteus flavescens	**Phyllostomidae**		yes														extant							
Artibeus anthonyi	**Phyllostomidae**	yes	yes								extinct													
Artibeus jamaicensis	**Phyllostomidae**					extant			extant	extant	extant	extant	extant	extant	extant		extant			extant	extant	extant	extant	extant
Artibeus lituratus	**Phyllostomidae**																							
Artibeus planirostris	**Phyllostomidae**																							
Artibeus schwartzi	**Phyllostomidae**		yes																					
Brachyphylla cavernarum	**Phyllostomidae**		yes																			extant	extant	extant
Brachyphylla nana nana	**Phyllostomidae**		yes						extinct		extant	extant	extant											
Brachyphylla nana pumila	**Phyllostomidae**		yes						extinct					extant			extinct							
Chiroderma improvisum	**Phyllostomidae**		yes																					
Cubanycteris silvai	**Phyllostomidae**	yes	yes								extinct													
Desmodus puntajudensis	**Phyllostomidae**	yes	yes								extinct													
Erophylla bombifrons	**Phyllostomidae**		yes											extant									extant	
Erophylla sezekorni	**Phyllostomidae**		yes	extant		extant	extant	extant	extant		extant	extant	extant				extant							
Glossophaga longirostris	**Phyllostomidae**																							
Glossophaga soricina	**Phyllostomidae**																extant							
Macrotus waterhousii	**Phyllostomidae**		yes	extant			extant	extant	extant	extant	extant	extant	extant	extant	extinct		extant		extant				extinct	
Monophyllus plethodon	**Phyllostomidae**		yes																				extinct	
Monophyllus redmani	**Phyllostomidae**		yes	extinct					extinct		extant	extant	extinct	extant	extinct		extant						extant	
Phyllonycteris aphylla	**Phyllostomidae**		yes														extant							
Phyllonycteris major	**Phyllostomidae**	yes	yes																				extinct	
Phyllonycteris poeyi	**Phyllostomidae**		yes	extinct					extinct		extant	extant		extant										
Phyllops falcatus	**Phyllostomidae**		yes						extant		extant	extinct	extant	extant										
Phyllops silvai	**Phyllostomidae**	yes	yes								extinct													
Phyllops vetus	**Phyllostomidae**	yes	yes								extinct													
Stenoderma rufum	**Phyllostomidae**		yes																				extant	extant

Species	Family	Extinct in West Indies	Endemic to West Indies	Great Abaco	Little Abaco	Mayaguana	East Plana Cay	San Salvador	Cayman Brac	Little Cayman	Cuba	Isle of Pines	Grand Cayman	Hispaniola	Ile de la Gonave	Ile de la Tortue	Jamaica	Mona	Navassa	Anegada	Culebra	Guana	Puerto Rico	St. John
				Bahamas	Bahamas	Bahamas	Bahamas	Bahamas	Greater Antilles	Greater Antilles	Greater Antilles	Greater Antilles	Greater Antilles	Greater Antilles	Greater Antilles	Greater Antilles	Greater Antilles	Greater Antilles	Greater Antilles	Greater Antilles	Greater Antilles	Greater Antilles	Greater Antilles	Greater Antilles
Sturnira lilium	**Phyllostomidae**																							
Sturnira thomasi	**Phyllostomidae**		yes																					
Tonatia saurophila	**Phyllostomidae**	yes															extinct							
Antrozous pallidus	**Vespertilionidae**										extant													
Eptesicus fuscus	**Vespertilionidae**			extant				extant	extant		extant	extant	extant	extant			extant						extant	
Eptesicus guadeloupensis	**Vespertilionidae**		yes																					
Lasiurus degelidus	**Vespertilionidae**		yes														extant							
Lasiurus insularis	**Vespertilionidae**		yes								extant													
Lasiurus intermedius	**Vespertilionidae**		yes								extant	extant		extinct										
Lasiurus minor	**Vespertilionidae**		yes			extant								extant									extant	
Lasiurus pfeifferi	**Vespertilionidae**		yes								extant													
Myotis cf. M. austroriparius	**Vespertilionida**	yes	yes	extinct																				
Myotis dominicensis	**Vespertilionidae**		yes																					
Myotis martiniquensis	**Vespertilionidae**		yes																					
Nycticeius cubanus	**Vespertilionidae**		yes								extant													
			**Extant # species**	**5**	**1**	**3**	**2**	**4**	**6**	**2**	**28**	**14**	**8**	**18**	**2**	**0**	**21**	**3**	**1**	**1**	**3**	**3**	**13**	**6**
			**Extinct # species**	**8**	**0**	**0**	**0**	**0**	**4**	**0**	**8**	**3**	**4**	**3**	**4**	**0**	**3**	**0**	**0**	**0**	**0**	**0**	**3**	**0**
			**Total species**	**13**	**1**	**3**	**2**	**4**	**10**	**2**	**36**	**17**	**12**	**21**	**6**	**0**	**24**	**3**	**1**	**1**	**3**	**3**	**16**	**6**

Species	Family	Extinct in West Indies	Endemic to West Indies	St. Thomas	Tortola	Vieques	Virgin Gorda	St. Croix	Anguilla	St. Barthelemy	St. Eustatius	St. Martin	Tintamarre	Antigua	Barbuda	Barbados	Dominica	Bequia	Carriacou	Mustique	Union	Guadeloupe	La Desirade
				**Greater Antilles**	**Greater Antilles**	**Greater Antilles**	**Greater Antilles**	**Greater Antilles**	**Lesser Antilles**	**Lesser Antilles**	**Lesser Antilles**	**Lesser Antilles**	**Lesser Antilles**	**Lesser Antilles**	**Lesser Antilles**	**Lesser Antilles**	**Lesser Antilles**	**Lesser Antilles**	**Lesser Antilles**	**Lesser Antilles**	**Lesser Antilles**	**Lesser Antilles**	**Lesser Antilles**
Eumops auripendulus	**Molossidae**																						
Eumops glaucinus	**Molossidae**																						
Eumops perotis	**Molossidae**																						
Molossus molossus	**Molossidae**			extant	extant	extant	extant	extant	extant	extant	extant	extant		extant	extant	extant	extant		extant		extant	extant	extant
Mormopterus minutus	**Molossidae**		yes																				
Nyctinomops laticaudatus	**Molossidae**																						
Nyctinomops macrotis	**Molossidae**																						
Tadarida brasiliensis	**Molossidae**								extant	extant	extant	extant		extant	extant		extant					extant	extant
Mormoops blainvillei	**Mormoopidae**		yes						extinct					extinct	extinct								
Mormoops magna	**Mormoopidae**	yes	yes																				
Mormoops megalophylla	**Mormoopidae**	yes																					
Pteronotus davyi	**Mormoopidae**																extant						
Pteronotus macleayii	**Mormoopidae**		yes																				
Pteronotus parnelli parnelliii	**Mormoopidae**		yes																				
Pteronotus parnellii portoricensis	**Mormoopidae**		yes											extinct									
Pteronotus parnellii pusillus	**Mormoopidae**		yes																				
Pteronotus parnellii rubiginosus	**Mormoopidae**																						
Pteronotus pristinus	**Mormoopidae**	yes	yes																				
Pteronotus quadridens	**Mormoopidae**		yes																				
Pteronotus sp. nov.	**Mormoopidae**	yes	yes																				
Chilonatalus micropus macer	**Natalidae**		yes																				
Chilonatalus micropus micropus	**Natalidae**		yes																				
Chilonatalus tumidifrons	**Natalidae**		yes																				
Natalus jamaicensis	**Natalidae**		yes																				
Natalus major	**Natalidae**		yes																				
Natalus primus	**Natalidae**	yes	yes																				
Natalus stramineus	**Natalidae**		yes						extant			extant		extant	extant		extant					extant	
Nyctiellus lepidus	**Natalidae**		yes																				
Noctilio leporinus	**Noctlilionidae**			extant		extant		extant				extant		extant	extant	extant	extant		extant			extant	
Ardops nichollsi	**Phyllostomidae**		yes								extant	extant					extant					extant	
Ariteus flavescens	**Phyllostomidae**		yes																				
Artibeus anthonyi	**Phyllostomidae**	yes	yes																				
Artibeus jamaicensis	**Phyllostomidae**			extant	extant	extant	extant	extant	extant	extant	extant	extant		extant	extant	extant	extant	extant	extant	extant	extant	extant	extant
Artibeus lituratus	**Phyllostomidae**																						
Artibeus planirostris	**Phyllostomidae**																						
Artibeus schwartzi	**Phyllostomidae**		yes													extant							
Brachyphylla cavernarum	**Phyllostomidae**		yes	extant				extant	extant	extant	extant	extant		extant	extant	extant	extant					extant	extant
Brachyphylla nana nana	**Phyllostomidae**		yes																				
Brachyphylla nana pumila	**Phyllostomidae**		yes																				
Chiroderma improvisum	**Phyllostomidae**		yes																			extant	
Cubanycteris silvai	**Phyllostomidae**	yes	yes																				
Desmodus puntajudensis	**Phyllostomidae**	yes	yes																				
Erophylla bombifrons	**Phyllostomidae**		yes																				
Erophylla sezekorni	**Phyllostomidae**		yes																				
Glossophaga longirostris	**Phyllostomidae**																		extant		extant		
Glossophaga soricina	**Phyllostomidae**																						
Macrotus waterhousii	**Phyllostomidae**		yes						extinct														
Monophyllus plethodon	**Phyllostomidae**		yes						extant	extant		extant		extant	extant	extant	extant					extant	
Monophyllus redmani	**Phyllostomidae**		yes																				
Phyllonycteris aphylla	**Phyllostomidae**		yes																				
Phyllonycteris major	**Phyllostomidae**	yes	yes																				
Phyllonycteris poeyi	**Phyllostomidae**		yes																				
Phyllops falcatus	**Phyllostomidae**		yes																				
Phyllops silvai	**Phyllostomidae**	yes	yes																				
Phyllops vetus	**Phyllostomidae**	yes	yes																				
Stenoderma rufum	**Phyllostomidae**		yes	extant		extant		extant															

Species	Family	Extinct in West Indies	Endemic to West Indies	St. Thomas	Tortola	Vieques	Virgin Gorda	St. Croix	Anguilla	St. Barthelemy	St. Eustatius	St. Martin	Tintamarre	Antigua	Barbuda	Barbados	Dominica	Bequia	Carriacou	Mustique	Union	Guadeloupe	La Desirade
				Greater Antilles	Greater Antilles	Greater Antilles	Greater Antilles	Greater Antilles	Lesser Antilles	Lesser Antilles	Lesser Antilles	Lesser Antilles	Lesser Antilles	Lesser Antilles	Lesser Antilles	Lesser Antilles	Lesser Antilles	Lesser Antilles	Lesser Antilles	Lesser Antilles	Lesser Antilles	Lesser Antilles	Lesser Antilles
Sturnira lilium	**Phyllostomidae**																extant						
Sturnira thomasi	**Phyllostomidae**		yes																			extant	
Tonatia saurophila	**Phyllostomidae**	yes																					
Antrozous pallidus	**Vespertilionidae**																						
Eptesicus fuscus	**Vespertilionidae**																extant						
Eptesicus guadeloupensis	**Vespertilionidae**		yes																			extant	
Lasiurus degelidus	**Vespertilionidae**		yes																				
Lasiurus insularis	**Vespertilionidae**		yes																				
Lasiurus intermedius	**Vespertilionidae**		yes																				
Lasiurus minor	**Vespertilionidae**		yes																				
Lasiurus pfeifferi	**Vespertilionidae**		yes																				
Myotis cf. M. austroriparius	**Vespertilionida(**	yes	yes																				
Myotis dominicensis	**Vespertilionidae**		yes														extant					extant	
Myotis martiniquensis	**Vespertilionidae**		yes													extant							
Nycticeius cubanus	**Vespertilionidae**		yes																				
			**Extant # species**	**5**	**2**	**4**	**2**	**5**	**6**	**5**	**5**	**8**	**0**	**7**	**7**	**7**	**12**	**1**	**4**	**1**	**3**	**12**	**4**
			**Extinct # species**	**0**	**0**	**0**	**0**	**0**	**2**	**0**	**0**	**0**	**0**	**2**	**1**	**0**	**0**	**0**	**0**	**0**	**0**	**0**	**0**
			**Total species**	**5**	**2**	**4**	**2**	**5**	**8**	**5**	**5**	**8**	**0**	**9**	**8**	**7**	**12**	**1**	**4**	**1**	**3**	**12**	**4**
