# Supplementary material for: Deglaciation explains bat extinction in the Caribbean
Source: Ecol Evol. 2012 Nov 6;2(12):3045–51. doi: 10.1002/ece3.399 (PMC3538999; doi:10.1002/ece3.399)
Supplement: Supplementary file 5 [file ece30002-3045-SD5.doc]

**Electronic Supplementary Materials**

**Supplementary Table S1**. Caribbean bat species inventory by island and archipelago.

**Supplementary Table S2**. Slopes and significance of SARs for the Bahamas after excluding *Artibeus jamaicensis* and accounting for coral accretion since the LGM.

| Archipelago | Period | Slope ± standard error | *R^2^* | *P*-value |
| --- | --- | --- | --- | --- |
| Bahamas | LGM | 0.35 ± 0.06 | 0.82 | 0.0011 |
|  | Present/LGM | 0.26 ± 0.02 | 0.83 | 0.0000 |

**Supplementary Table S3**. Analyses of covariance (ANCOVA) testing for the homogeneity of intercepts and slopes of SARs at LGM and present for the Bahamas after excluding *Artibeus jamaicensis* and accounting for coral accretion since the LGM.

| Archipelago | Time period as factor | *P*-value | Interaction Log Area & Time | *P*-value |
| --- | --- | --- | --- | --- |
| Bahamas | 0.412 ± 0.308 | 0.1923 | -0.113 ± 0.097 | 0.2551 |

**Supplementary Figure S4**. Species-area curves and observed vs. predicted richness for the Bahamas at the LGM and present after excluding *Artibeus jamaicensis* and accounting for coral accretion since the LGM. Shaded areas indicate the 95% confidence interval around the mean of the curves. Left: SARs fitted to observed current and estimated LGM values. Right: predicted vs. observed species richness. The curve of slope = 1 indicates where the LGM SAR perfectly predicts current species richness. The LGM SAR underestimates current species richness in the area below the curve, and overestimates current richness in the area above the curve.
